# Supplementary material for: Replicon-based genome-wide CRISPR knockout screening for the identification of host factors involved in viral replication
Source: Nat Commun. 2025 Dec 10;16:11028. doi: 10.1038/s41467-025-65979-3 (PMC12696002; doi:10.1038/s41467-025-65979-3)
Supplement: Supplementary file 1 — Supplementary information [file 41467_2025_65979_MOESM1_ESM.pdf]

Supplementary Information for:

Replicon-based genome-wide CRISPR knockout screening for the identification of host factors involved in viral replication

Karen W. Cheng[1], Madhura Bhave[1], Andrew L. Markhard[1,3], Duo Peng[1], Karan D. Bhatt[1,4], Katherine Travisano[1,5], Josette V. Medicielo[1,6], Astrid Anaya[1], Sanae Lembirik[2], Leila Njoya[1], Manu Anantpadma[2], Jens H. Kuhn[2], Andreas S. Puschnik[1], Amy Kistler[1*]

[1]Chan Zuckerberg Biohub, San Francisco, CA, US, [2]Integrated Research Facility at Fort Detrick, Division of Clinical Research, National Institute of Allergy and Infectious Diseases, National Institutes of Health, Fort Detrick, Frederick, MD, USA, [3]Present address: New York University Grossman School of Medicine, New York, NY, USA, [4]Present address: Washington University School of Medicine, St. Louis, MO, USA, [5]Present address: Stanford University, Palo Alto, CA, USA, [6]Present address: Medical College of Wisconsin, Milwaukee, WI, USA.

This PDF file includes:

Supplementary Figures **1 - 5**

Supplementary Figure 1: Dengue virus type 2 replicon cell line design. 1

Supplementary Figure 2: Genetic confirmation of gene KOs from DENV-2 replicon screen. 2

Supplementary Figure 3: CHIKV replicon design and confirmation of gene KOs. 3

Supplementary Figure 4: EBOV minigenome replicon system overview. 4

Supplementary Figure 5: Selection and. Validation of EBOV replicon screen hits. 5

Supplementary Notes **6 - 7**

Supplementary Note 1: Complementation experiments summary 6

Supplementary Note 2: Gating strategy for genome-wide CRISPR KO screen FACS. 7

Supplementary References **8**

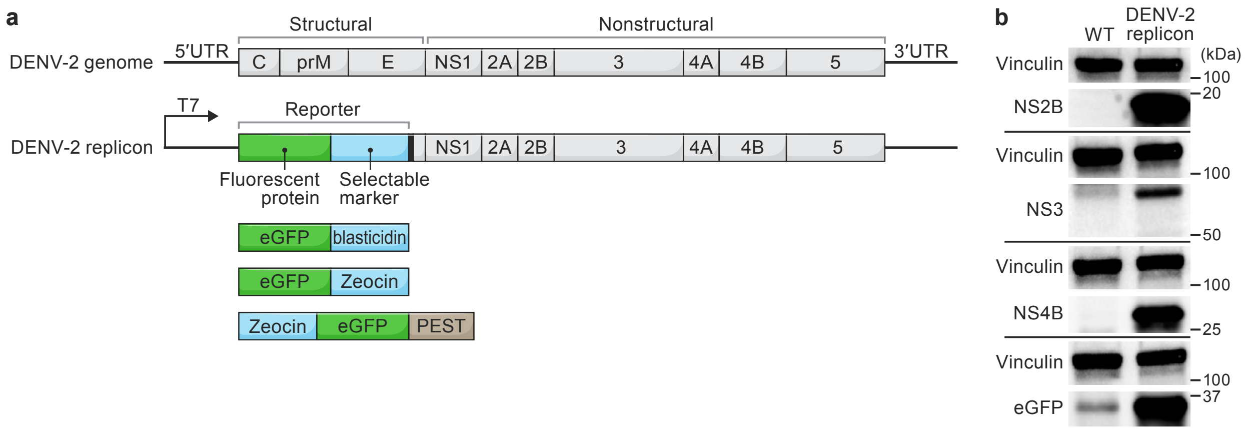

Supplementary Figure 1. Dengue virus type 2 replicon (DENV-2) cell line design. **a**, Diagram of the dengue virus type 2 (DENV-2) 16681 genome. Untranslated regions (UTRs) and the structural and nonstructural genes in the genome are labeled. **b**, Schematic diagram of the DENV-2 16681 replicon. The T7 promoter used for *in vitro* transcription is indicated with an arrow. Structural genes are replaced with a fluorescent protein reporter (green)-selectable marker (light blue) gene fusion cassette. Different reporter-selection cassettes tested in this study: eGFP-blasticidin, eGFP-Zeocin, Zeocin-eGFP-PEST. **c**, Western blot confirmation of expression of DENV-2 proteins NS2B, NS3, NS4B, and eGFP reporter in the Huh7.5.1-Cas9 DENV-2 replicon cell line compared to the parental Huh7.5.1–Cas9 cell line. Loading control: vinculin (data shown are representative results from ≥ 2 replicates). Source data are provided as a Source Data file.

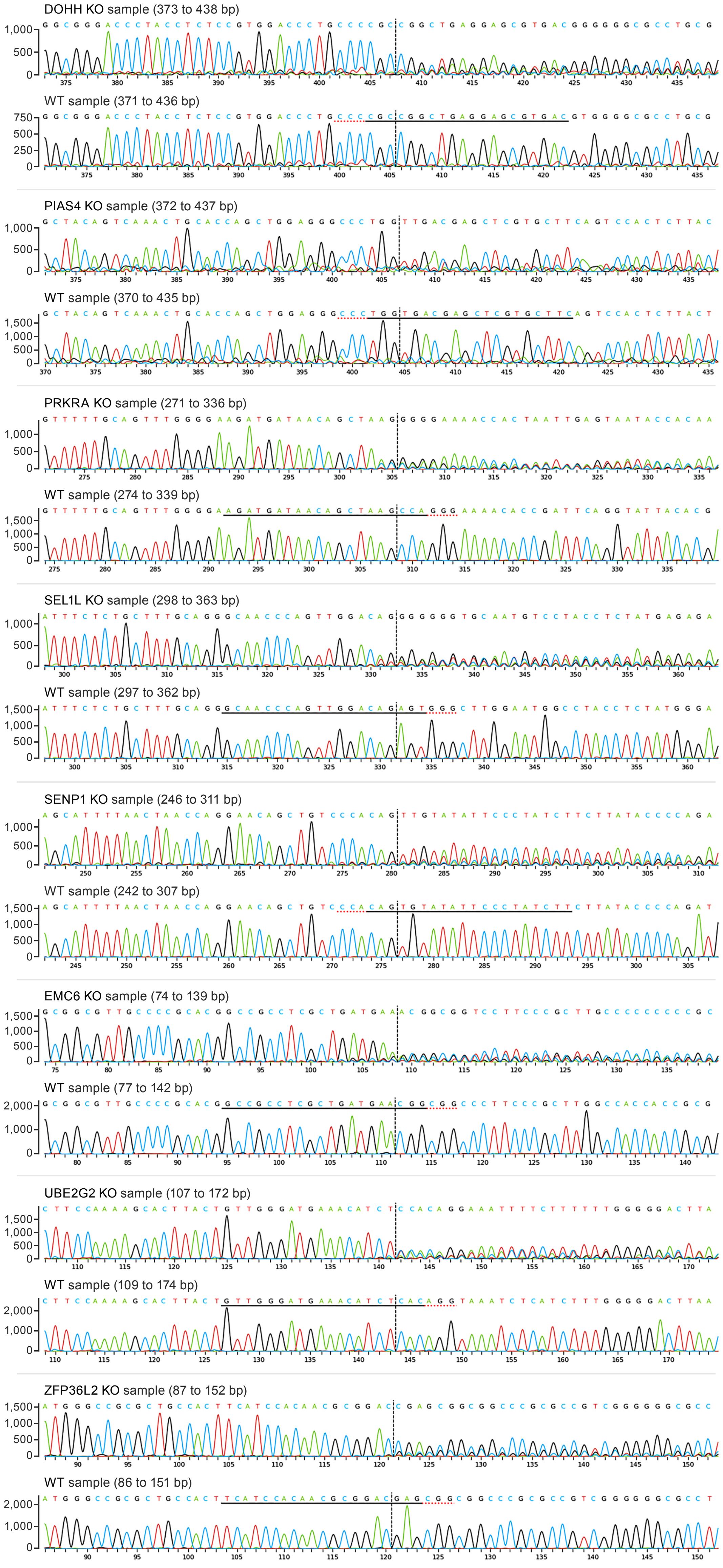

Supplementary Figure 2. Genetic confirmation of gene KOs from DENV-2 replicon screen. Sanger sequencing traces of target regions PCR amplified from genomic DNA extracted from wildtype and knockout (KO) cell line populations (n = 1 replicate).

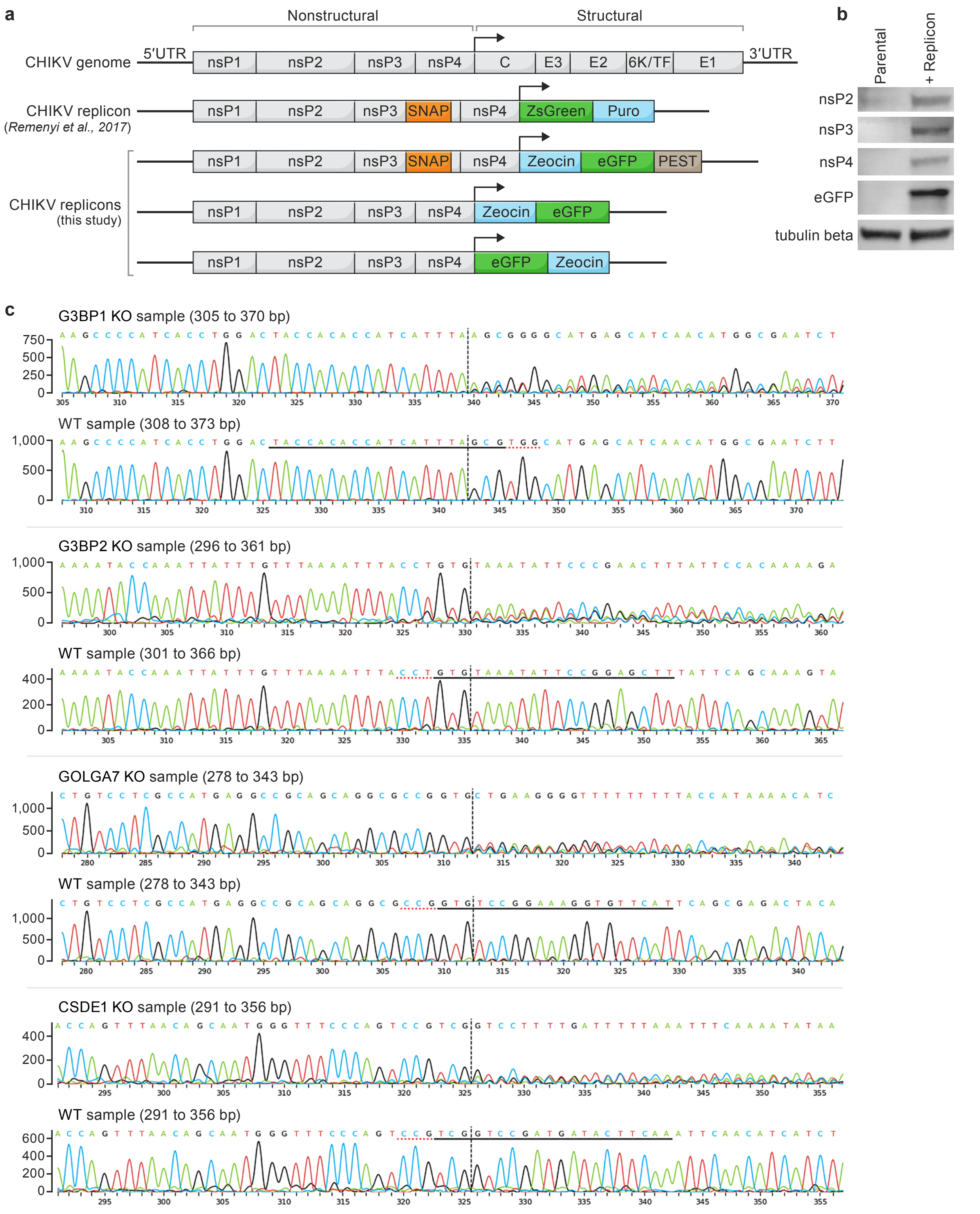

Supplementary Figure 3. CHIKV replicon design and confirmation of gene knockout KOs. **a**, CHIKV genome schematic (top) with diagrams of previously established nsP3-SNAP-tagged zsGreen-puromycin CHIKV replicon[1] with the replicon variants generated and tested in this study shown below. **b**, Western blot confirmation of expression of CHIKV nonstructural proteins nSP2, nSP3, and nSP4 and the eGFP reporter in the Huh7.5.1–Cas9 CHIKV replicon cell line compared to the parental Huh7.5.1–Cas9 cell line. Loading control: beta tubulin (data shown are representative results from $\geq$ 2 replicates). **c**, Genetic confirmation of gene targeting in independently generated populations of Huh7.5.1 knockout (KO) cell lines. Sanger sequencing traces of target regions amplified from genomic DNA extracted from wildtype and KO cell line populations (n = 1 replicate). Source data are provided as a Source Data file.

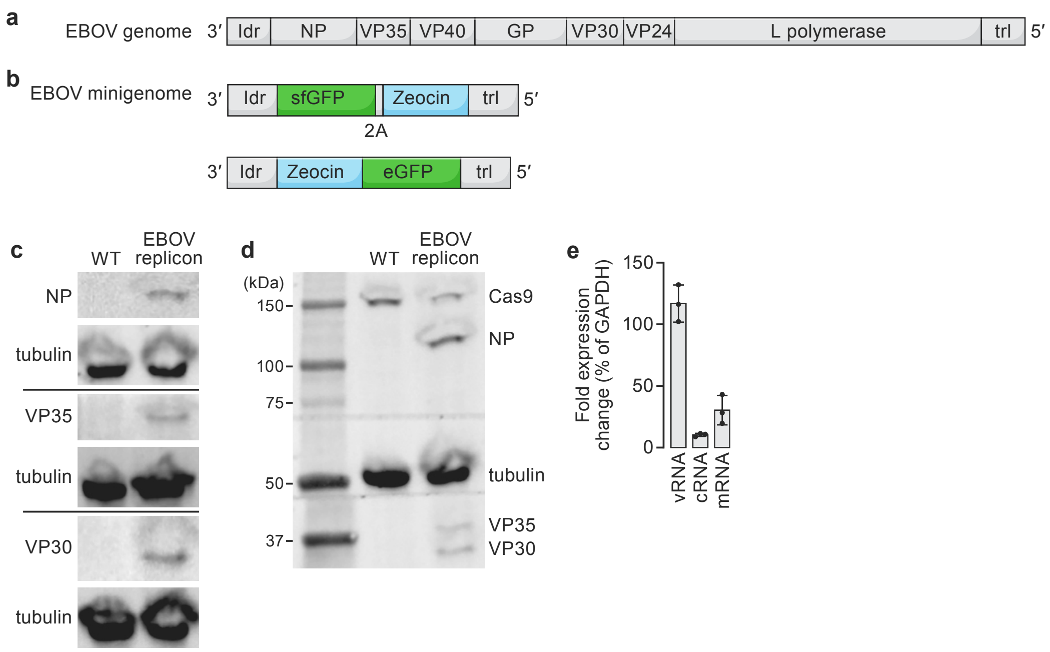

Supplementary Figure 4. EBOV minigenome replicon system overview. **a**, Diagram of Ebola virus (EBOV) genome structure. **b**, EBOV minigenome replicon variants tested for this study. **c**, Western blot showing the expression of 4cis proteins in the replicon cells. The cell lysates for replicon cells and control cells were probed with anti-NP, anti-VP35 and anti-VP30 antibodies. Parental Huh7.5.1-Cas9 (WT) cells were analyzed in parallel; loading control: beta tubulin . **d**, Western blot with P2A antibodies to assess expression and processing of the 4cis proteins harboring a P2A tag (NP, VP35, and VP30, and the integrated Cas9 gene) detectable in the parental Huh7.5.1–Cas9 cell line and the stable Huh7.5.1-Cas9 EBOV replicon cell line; loading control: beta tubulin. **e**, Relative expression levels of the three different types of minigenome RNA products expected in the replicon cell line. Total cellular RNA was isolated from replicon cells and used as an input for a strand-specific reverse transcription quantitative PCR (RT-qPCR) assay designed to detect the negative-sense minigenome viral RNA (vRNA), the positive-sense RNA transcript complementary to the vRNA that is generated during viral replication (cRNA), and the positive-sense messenger RNA (mRNA) that is transcribed from the minigenome vRNA template. The values plotted here are mean fold expression changes ± standard deviation for three independent technical replicates of RT-qPCR analyses for vRNA, mRNA and cRNA relative to the housekeeping control gene glyceraldehyde-3-phosphate dehydrogenase (GAPDH). Western blot data shown are representative results from ≥ 2 replicates. Source data are provided as a Source Data file.

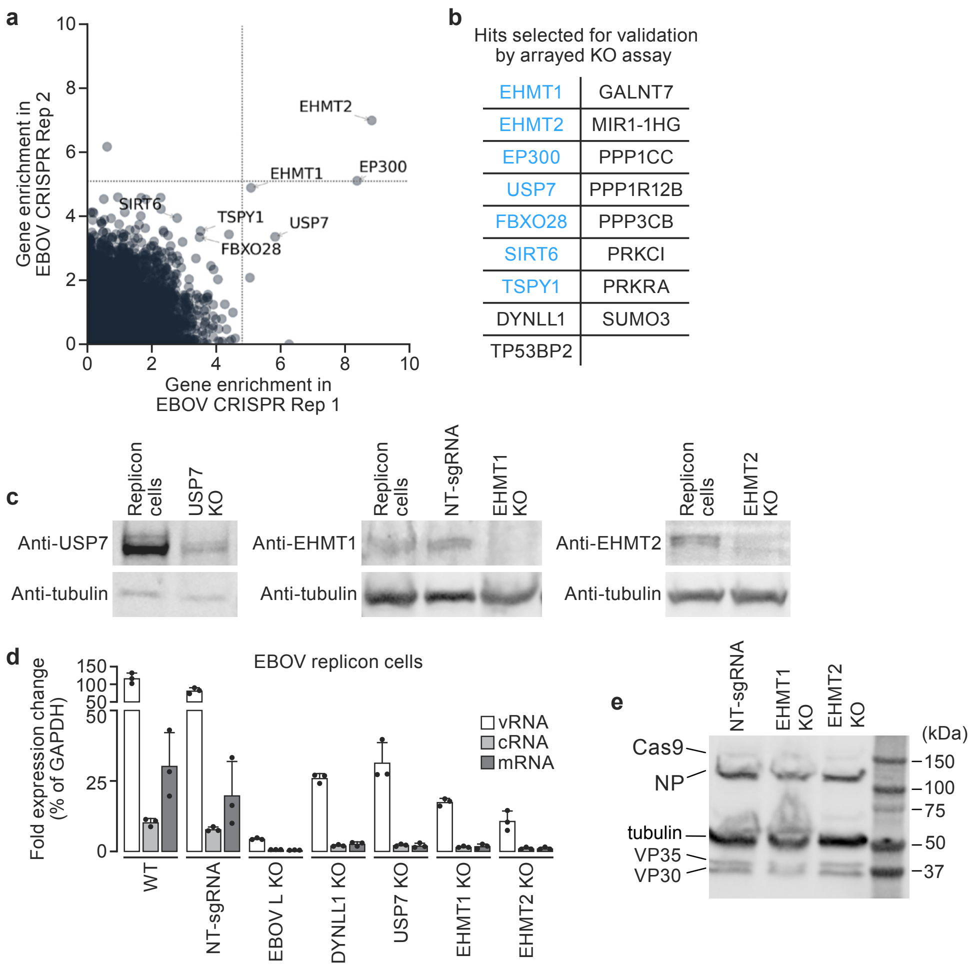

Supplementary Figure 5. Selection and validation of EBOV replicon screen hits. **a**, Correlation of hits across the two independent biological replicates of the CRISPR screen with the EBOV replicon cell line. **b**, List of hits selected for validation using an arrayed knockout (KO) assay. Blue text, common genes enriched across the two independent biological replicates; black text, genes among the top 200 enriched hits in either replicate of the screen that have been implicated to play a possible role in EBOV replication and transcription. **c**, Western blot validation of depletion of host factors EHMT1, EHMT2, and USP7 in the KO cell lines. Cell pellets collected from KO cells were lysed and probed with specific antibodies to assess protein levels in parental and KO replicon cell lines, with beta tubulin serving as a loading control. **d**, Relative ratios of viral RNA (vRNA), messenger RNA (mRNA), and RNA complementary to vRNA (cRNA) in the different knockout cell lines. Fold expression changes in vRNA, mRNA and cRNA relative to housekeeping control gene glyceraldehyde phosphate dehydrogenase (GAPDH). Values plotted correspond to mean ± SD for three independent technical replicates. **e**, Western blot analysis of the EBOV 4cis protein expression in independently generated EHMT1 and EHMT2 KO cell lines transiently co-transfected with the EBOV 4cis plasmid and a secreted nano luciferase minigenome plasmid. Cell pellets collected from NT, EHMT1 and EHMT2 KO cells 48 h post-transfection were lysed and probed with anti-2A antibodies to assess EBOV NP, VP35, and VP30 levels in the NT and KO cell lines; beta tubulin antibodies were applied as loading control. Western blot data shown are representative results from $\geq$ 2 replicates. Source data are provided as a Source Data file.

Supplementary Note 1: Complementation experiments summary

Several iterations of complementation experiments were deployed to try to further validate the role of gene hits from the CHIKV and EBOV screens. For each gene KO, overexpression CMV promoter-driven cDNAs with synonymous mutations in the PAM site were cloned into a lentiviral vector that co-expressed the fluorescent protein mCherry. With these constructs, generation of stable addback cell lines was feasible for constructs with smaller cDNAs (i.e., cDNAs less than < 2,000 bp in length: G3BP1, G3BP2, and GOLGA7), but repeated attempts for larger cDNAs (i.e., the cDNAs > 2,000bp in length: CSDE1, EHMT1, EHMT2, and USP7) failed, likely due to poor lentiviral packaging efficiency.

Using the available add-back lines, we tested replication of a CHIKV eGFP-zeocin replicon in the parental Huh7.5.1-Cas9 cells, the corresponding KO cells, and paired addback cell lines through transient transfection of the *in vitro* transcribed CHIKV replicon RNA. G3BP2 complementation clearly restored GFP expression to near-control levels (see Figure below), supporting on-target activity.

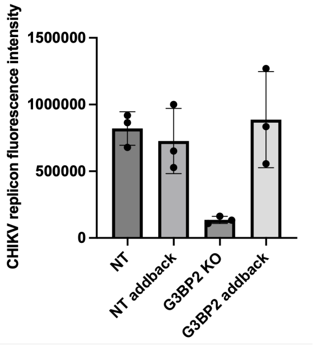

In contrast, the G3BP1 and GOLGA7 add-backs showed poor growth and no rescue, which we believe could reflect toxicity from strong promoter-driven over-expression. While we achieved robust rescue for G3BP2, we acknowledge that there are technical limitations for larger or dosage-sensitive genes. Further experiments involving lentiviral vectors with complementing cDNA constructs for all gene hits driven by a weaker promoter (SFFV), as well as lentiviral dilutions were attempted to mitigate possible cell toxicity effects from over-expression and yielded similar results. Alternative strategies, such as inducible promoters, may be needed to fully resolve complementation for those cases.

Supplementary Note 2: Gating strategy for genome-wide CRISPR KO screen FACS.

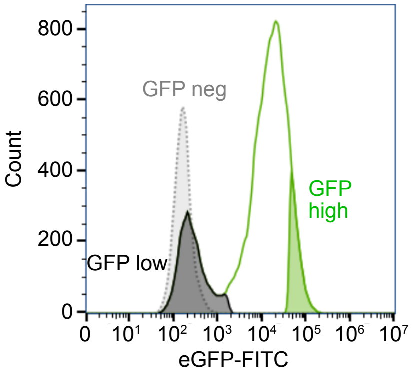

Gating strategy for genome-wide CRISPR KO screen FACS. Pooled eGFP-expressing replicon screen cells (20 days post transduction with the Human Brunello CRISPR knockout pooled sgRNA lentivirus library) were sorted into "eGFP-low" (bottom 20%, shaded dark gray) and eGFP-high (top 10%, shaded green) sub-populations using the parental Huh7.5.1.-Cas9-hygro cell line as a gating control ("eGFP-neg", shaded light gray).

Supplementary References

1. Remenyi, R., Roberts, G. C., Zothner, C., Merits, A. & Harris, M. SNAP-tagged Chikungunya Virus Replicons Improve Visualisation of Non-Structural Protein 3 by Fluorescence Microscopy. *Sci Rep* **7**, 5682 (2017).
