# Supplementary material for: Replicon-based genome-wide CRISPR knockout screening for the identification of host factors involved in viral replication
Source: Nat Commun. 2025 Dec 10;16:11028. doi: 10.1038/s41467-025-65979-3 (PMC12696002; doi:10.1038/s41467-025-65979-3)
Supplement: Supplementary file 3 — Description of Additional Supplementary Files [file 41467_2025_65979_MOESM3_ESM.pdf]

Description of Additional Supplementary Files

Title: Supplementary Data 1

Description: Model-based Analysis of Genome-wide CRISPR/Cas9 Knockout (MAGeCK) robust ranking aggregation (RRA) results summary for each of the described genome-wide replicon screens (dengue virus 2 [DENV2], chikungunya virus [CHIKV], and Ebola virus [EBOV]).

Title: Supplementary Data 2

Description: Summary of the sgRNAs used for phenotypic analysis of independent gene knockouts.

Title: Supplementary Data 3

Description: Summary of plasmid constructs used in this study
