# Supplementary material for: Replicon-based genome-wide CRISPR knockout screening for the identification of host factors involved in viral replication
Source: Nat Commun. 2025 Dec 10;16:11028. doi: 10.1038/s41467-025-65979-3 (PMC12696002; doi:10.1038/s41467-025-65979-3)
Supplement: Supplementary file 7 — Reporting Summary [file 41467_2025_65979_MOESM7_ESM.pdf]

nature portfolio

Corresponding author(s): Amy L. Kistler

Last updated by author(s): Sep 2, 2025

Reporting Summary

Nature Portfolio wishes to improve the reproducibility of the work that we publish. This form provides structure for consistency and transparency in reporting. For further information on Nature Portfolio policies, see our Editorial Policies and the Editorial Policy Checklist.

Statistics

For all statistical analyses, confirm that the following items are present in the figure legend, table legend, main text, or Methods section.

n/a	Confirmed	
☐	☒	The exact sample size (*n*) for each experimental group/condition, given as a discrete number and unit of measurement
☐	☒	A statement on whether measurements were taken from distinct samples or whether the same sample was measured repeatedly
☐	☒	The statistical test(s) used AND whether they are one- or two-sided<br>*Only common tests should be described solely by name; describe more complex techniques in the Methods section.*
☐	☒	A description of all covariates tested
☐	☒	A description of any assumptions or corrections, such as tests of normality and adjustment for multiple comparisons
☐	☒	A full description of the statistical parameters including central tendency (e.g. means) or other basic estimates (e.g. regression coefficient) AND variation (e.g. standard deviation) or associated estimates of uncertainty (e.g. confidence intervals)
☐	☒	For null hypothesis testing, the test statistic (e.g. *F*, *t*, *r*) with confidence intervals, effect sizes, degrees of freedom and *P* value noted<br>*Give P values as exact values whenever suitable.*
☒	☐	For Bayesian analysis, information on the choice of priors and Markov chain Monte Carlo settings
☒	☐	For hierarchical and complex designs, identification of the appropriate level for tests and full reporting of outcomes
☐	☒	Estimates of effect sizes (e.g. Cohen's *d*, Pearson's *r*), indicating how they were calculated

Our web collection on statistics for biologists contains articles on many of the points above.

Software and code

Policy information about availability of computer code

Data collection	No software was used for data collection for this study.
Data analysis	Demultiplexed FASTQ files were analyzed using the 'count' subcommand of MAGeCK software (v0.5.9.4) to quantify gRNA abundance by matching reads to the gRNA library sequences, with the normalization method set to 'total'. The gRNA count tables were subsequently analyzed using the ‘test’ subcommand (to calculate the robust rank aggregation [RRA]) of MAGeCK software v0.5.9.4 to provide positive enrichment scores for each gene. The two-step process was automated using Nextflow v21.10.6. The analysis workflow standardizes metadata conventions.The computer code for this workflow is available at https://github.com/czbiohub-sf/CRISPRflow and permanently archived under DOI https://doi.org/10.5281/zenodo.15595933.<br><br>Enrichment of Gene Ontology (GO) terms among candidate gene hits from the DENV-2 replicon screen was performed with clusterProfiler (Bioconductor version Release [3.21], archived under DOI 10.18129/B9.bioc.clusterProfiler).<br><br>The open-source software program protospaceJAM (https://protospacejam.czbiohub.org/) was used to design genotyping primers targeting a 300-bp region around the predicted genomic cut site of each gRNA in the Brunello library for downstream PCR and sequence analysis of these sites.<br><br>Purified PCR products were submitted for Sanger sequencing and analyzed using Synthego ICE analysis software (https://ice.synthego.com/.

For manuscripts utilizing custom algorithms or software that are central to the research but not yet described in published literature, software must be made available to editors and reviewers. We strongly encourage code deposition in a community repository (e.g. GitHub). See the Nature Portfolio guidelines for submitting code & software for further information.

Data

Policy information about availability of data

All manuscripts must include a data availability statement. This statement should provide the following information, where applicable:

- Accession codes, unique identifiers, or web links for publicly available datasets
- A description of any restrictions on data availability
- For clinical datasets or third party data, please ensure that the statement adheres to our policy

Data that support the findings of this study have been deposited in the NCBI Gene Expression Omnibus (GEO) repository (https://www.ncbi.nlm.nih.gov/geo/) with the GEO study accession GSE284379 and the NCBI sequence read archive (SRA) repository (https://www.ncbi.nlm.nih.gov/sra) with the SRA project accession PRJNA1198907, and the primary sample accessions GSE284379 - GSM8683017.

Research involving human participants, their data, or biological material

Policy information about studies with human participants or human data. See also policy information about sex, gender (identity/presentation), and sexual orientation and race, ethnicity and racism.

Reporting on sex and gender	*Use the terms sex (biological attribute) and gender (shaped by social and cultural circumstances) carefully in order to avoid confusing both terms. Indicate if findings apply to only one sex or gender; describe whether sex and gender were considered in study design; whether sex and/or gender was determined based on self-reporting or assigned and methods used. Provide in the source data disaggregated sex and gender data, where this information has been collected, and if consent has been obtained for sharing of individual-level data; provide overall numbers in this Reporting Summary. Please state if this information has not been collected. Report sex- and gender-based analyses where performed, justify reasons for lack of sex- and gender-based analysis.*
Reporting on race, ethnicity, or other socially relevant groupings	*Please specify the socially constructed or socially relevant categorization variable(s) used in your manuscript and explain why they were used. Please note that such variables should not be used as proxies for other socially constructed/relevant variables (for example, race or ethnicity should not be used as a proxy for socioeconomic status). Provide clear definitions of the relevant terms used, how they were provided (by the participants/respondents, the researchers, or third parties), and the method(s) used to classify people into the different categories (e.g. self-report, census or administrative data, social media data, etc.) Please provide details about how you controlled for confounding variables in your analyses.*
Population characteristics	*Describe the covariate-relevant population characteristics of the human research participants (e.g. age, genotypic information, past and current diagnosis and treatment categories). If you filled out the behavioural & social sciences study design questions and have nothing to add here, write "See above."*
Recruitment	*Describe how participants were recruited. Outline any potential self-selection bias or other biases that may be present and how these are likely to impact results.*
Ethics oversight	*Identify the organization(s) that approved the study protocol.*

Note that full information on the approval of the study protocol must also be provided in the manuscript.

Field-specific reporting

Please select the one below that is the best fit for your research. If you are not sure, read the appropriate sections before making your selection.

☒ Life sciences ☐ Behavioural & social sciences ☐ Ecological, evolutionary & environmental sciences

For a reference copy of the document with all sections, see nature.com/documents/nr-reporting-summary-flat.pdf

Life sciences study design

All studies must disclose on these points even when the disclosure is negative.

Sample size	CRISPR KO screens: Approximately 40-60 million cells were used and collected in each replicate of genome-wide CRISPR knockout screens described in this study in order to obtain >500X coverage of the Human Brunello CRISPR knockout pooled sgRNA library (76,441 distinct gRNAs, targeting 19,114 genes).<br><br>Flow cytometry experiments: in general a minimum of 1,000 cells and maximum o 10,000 cells were analyzed for each sample.
Data exclusions	No data were excluded from the analysis
Replication	For each genome-wide screen we grew up approximately 160 million stable replicon cells, then split them into duplicate sets of flasks that were processed separately and independently (including transduction with the Brunello sgRNA lentivirus library, selection on puromycin, followed by passaging for phenotype development, and subsequent cell sorting).<br><br>For subsequent follow-up experiments, a minimum of 2 technical replicates were performed for replicon screen phenotype validation; for RT-

	qPCR a minimum of 3 technical replicates was performed for each sample. Where feasible, biological replicates were also performed. Western blot experiments were performed at least 2 times for all representative results shown in the manuscript.
Randomization	In this study, no randomization was employed. The primary comparisons were between cell lines harboring gene knockouts (KOs) and control cells that did not harbor gene KOs. For this study, we utilized both the parental cell line and the "NT" cell line, a cell line transduced with an empty (non-targeting sgRNA) lentivirus in parallel with the KO cell lines as the baseline controls.
Blinding	As this study focused on comparing KO cell phenotypes to control (WT or NT cell), blinding was not implemented.

Reporting for specific materials, systems and methods

We require information from authors about some types of materials, experimental systems and methods used in many studies. Here, indicate whether each material, system or method listed is relevant to your study. If you are not sure if a list item applies to your research, read the appropriate section before selecting a response.

Materials & experimental systems

n/a	Involved in the study	
☐	☒	Antibodies
☐	☒	Eukaryotic cell lines
☐	☐	Palaeontology and archaeology
☐	☐	Animals and other organisms
☐	☐	Clinical data
☐	☐	Dual use research of concern
☐	☐	Plants

Methods

n/a	Involved in the study	
☐	☐	ChIP-seq
☐	☒	Flow cytometry
☐	☐	MRI-based neuroimaging

Antibodies

Antibodies used	DENV NS2B (Genetex, GTX124246), DENV NS3 (Genetex, GTX629477), DENV NS4B (GeneTex, GTX103349), GFP (GeneTex, GTX113617), vinculin 7F9 (Santa Cruz Biotechnology, sc-73614), CHIKV nsP2 (Genetex, GTX135188), CHIKV nsP3 (Genetex, GTX135189), CHIKV nsP4 (Thermo Fisher Scientific, PA5-117443), beta tubulin (Cell Signaling, 15115 and Thermo Fisher Scientific, MA5-16308), NP (IBT Bioservices, 0301-012), EBOV VP35 (Kerafast, Kf Ab02366-1.1), EBOV VP30 (GeneTex, GTX134035), 2A (Novus, NBP2-59627), EHMT1 (Abcam, ab241306), EHMT2 (Thermo Fisher Scientific, MA5-14880), USP7 (Thermo Fisher Scientific, PA5-34911).
Validation	DENV2 NS2B (Genetex, GTX124246; https://www.genetex.com/Product/Detail/Dengue-virus-NS2B-protein-antibody/GTX124246), DENV NS3 (Genetex GTX629477, https://www.genetex.com/Product/Detail/Dengue-virus-NS3-protein-antibody-GT2811/GTX629477), DENV NS4B (Genetex GTX103349, https://www.genetex.com/Product/Detail/Dengue-virus-NS4B-protein-antibody/GTX103349), GFP (Genetex GTX113617, https://www.genetex.com/Product/Detail/GFP-antibody/GTX113617), Vinculin 7F9 (Santa Cruz Biotechnology sc-73614, https://www.citeab.com/antibodies/829913-sc-73614-vinculin-antibody-7f9), CHIKV nsP2 (Genetex GTX135188, https://www.genetex.com/Product/Detail/Chikungunya-virus-nsP2-antibody/GTX135188), CHIKV nsP3 (Genetex GTX135189, https://www.genetex.com/Product/Detail/Chikungunya-virus-nsP3-antibody/GTX135189), CHIKV nsP4 (Thermo Fisher Scientific PA5-117443, https://www.thermofisher.com/antibody/product/Chikungunya-Virus-nsP4-Antibody-Polyclonal/PA5-117443), beta tubulin (Cell signaling 15115, https://www.cellsignal.com/products/primary-antibodies/b-tubulin-d2n5g-rabbit-mab/15115; Thermo Fisher Scientific MA5-16308, https://www.thermofisher.com/antibody/product/beta-Tubulin-Loading-Control-Antibody-clone-BT7R-Monoclonal/MA5-16308), EBOV NP (IBT Bioservices 0301-012, https://www.thomassci.com/p/rabbit-anti-ebov-np-pab), EBOV VP35 (Kerafast Kf EMS703, https://www.kerafast.com/item/1009/anti-zaire-ebola-virus-vp35-protein-n-terminal-10c7-antibody), EBOV VP30 (Genetex GTX134035, genetex.com/Product/Detail/Ebola-virus-VP30-antibody/GTX134035), 2A (Novus NBP2-59627, https://www.novusbio.com/products/2a-peptide-antibody-3h4_nbp2-59627), EHMT1 (Abcam ab241306, https://www.abcam.com/en-us/products/primary-antibodies/ehmt1-glp-antibody-ab241306), EHMT2 (Thermo Fisher Scientific MA5-14880, https://www.thermofisher.com/antibody/product/EHMT2-Antibody-clone-B-133-9-Monoclonal/MA5-14880), USP7 (Thermo Fisher Scientific PA5-34911, https://www.thermofisher.com/antibody/product/USP7-Antibody-Polyclonal/PA5-34911)

Eukaryotic cell lines

Policy information about cell lines and Sex and Gender in Research

Cell line source(s)	Grivet (Chlorocebus aethiops) kidney epithelial Vero E6 cells were sourced from the American Type Culture Collection (ATCC; #CRL-1586) by BEI Resources and specially banked by Lonza for the Integrated Research Facility at Fort Detrick (IRF-Frederick). Human hepatocyte-derived carcinoma Huh7.5.1 cells were sourced from Apath (Dr. Charles M. Rice) and Scripps Research (Dr. Francis V. Chisari). Human embryonic kidney (HEK) epithelial 293FT cells used to generate lentiviruses were a generous gift from the Jan Carette lab (Stanford University).
Authentication	None of the cell line sources were authenticated
Mycoplasma contamination	Cell lines were not tested for Mycoplasma contamination.
Commonly misidentified lines (See ICLAC register)	*Name any commonly misidentified cell lines used in the study and provide a rationale for their use.*

Palaeontology and Archaeology

Specimen provenance	*Provide provenance information for specimens and describe permits that were obtained for the work (including the name of the issuing authority, the date of issue, and any identifying information). Permits should encompass collection and, where applicable, export.*
Specimen deposition	*Indicate where the specimens have been deposited to permit free access by other researchers.*
Dating methods	*If new dates are provided, describe how they were obtained (e.g. collection, storage, sample pretreatment and measurement), where they were obtained (i.e. lab name), the calibration program and the protocol for quality assurance OR state that no new dates are provided.*

☐ Tick this box to confirm that the raw and calibrated dates are available in the paper or in Supplementary Information.

Ethics oversight	*Identify the organization(s) that approved or provided guidance on the study protocol, OR state that no ethical approval or guidance was required and explain why not.*

Note that full information on the approval of the study protocol must also be provided in the manuscript.

Animals and other research organisms

Policy information about studies involving animals; ARRIVE guidelines recommended for reporting animal research, and Sex and Gender in Research

Laboratory animals	*For laboratory animals, report species, strain and age OR state that the study did not involve laboratory animals.*
Wild animals	*Provide details on animals observed in or captured in the field; report species and age where possible. Describe how animals were caught and transported and what happened to captive animals after the study (if killed, explain why and describe method; if released, say where and when) OR state that the study did not involve wild animals.*
Reporting on sex	*Indicate if findings apply to only one sex; describe whether sex was considered in study design, methods used for assigning sex. Provide data disaggregated for sex where this information has been collected in the source data as appropriate; provide overall numbers in this Reporting Summary. Please state if this information has not been collected. Report sex-based analyses where performed, justify reasons for lack of sex-based analysis.*
Field-collected samples	*For laboratory work with field-collected samples, describe all relevant parameters such as housing, maintenance, temperature, photoperiod and end-of-experiment protocol OR state that the study did not involve samples collected from the field.*
Ethics oversight	*Identify the organization(s) that approved or provided guidance on the study protocol, OR state that no ethical approval or guidance was required and explain why not.*

Note that full information on the approval of the study protocol must also be provided in the manuscript.

Clinical data

Policy information about clinical studies

All manuscripts should comply with the ICMJE guidelines for publication of clinical research and a completed CONSORT checklist must be included with all submissions.

Clinical trial registration	*Provide the trial registration number from ClinicalTrials.gov or an equivalent agency.*
Study protocol	*Note where the full trial protocol can be accessed OR if not available, explain why.*
Data collection	*Describe the settings and locales of data collection, noting the time periods of recruitment and data collection.*
Outcomes	*Describe how you pre-defined primary and secondary outcome measures and how you assessed these measures.*

Dual use research of concern

Policy information about dual use research of concern

Hazards

Could the accidental, deliberate or reckless misuse of agents or technologies generated in the work, or the application of information presented in the manuscript, pose a threat to:

No	Yes	
☒	☐	Public health
☒	☐	National security
☒	☐	Crops and/or livestock
☒	☐	Ecosystems
☒	☐	Any other significant area

Experiments of concern

Does the work involve any of these experiments of concern:

No	Yes	
☒	☐	Demonstrate how to render a vaccine ineffective
☒	☐	Confer resistance to therapeutically useful antibiotics or antiviral agents
☒	☐	Enhance the virulence of a pathogen or render a nonpathogen virulent
☒	☐	Increase transmissibility of a pathogen
☒	☐	Alter the host range of a pathogen
☒	☐	Enable evasion of diagnostic/detection modalities
☒	☐	Enable the weaponization of a biological agent or toxin
☒	☐	Any other potentially harmful combination of experiments and agents

Plants

Seed stocks

Report on the source of all seed stocks or other plant material used. If applicable, state the seed stock centre and catalogue number. If plant specimens were collected from the field, describe the collection location, date and sampling procedures.

Novel plant genotypes

Describe the methods by which all novel plant genotypes were produced. This includes those generated by transgenic approaches, gene editing, chemical/radiation-based mutagenesis and hybridization. For transgenic lines, describe the transformation method, the number of independent lines analyzed and the generation upon which experiments were performed. For gene-edited lines, describe the editor used, the endogenous sequence targeted for editing, the targeting guide RNA sequence (if applicable) and how the editor was applied.

Authentication

Describe any authentication procedures for each seed stock used or novel genotype generated. Describe any experiments used to assess the effect of a mutation and, where applicable, how potential secondary effects (e.g. second site T-DNA insertions, mosiacism, off-target gene editing) were examined.

ChIP-seq

Data deposition

☐ Confirm that both raw and final processed data have been deposited in a public database such as GEO.

☐ Confirm that you have deposited or provided access to graph files (e.g. BED files) for the called peaks.

Data access links
May remain private before publication.

For "Initial submission" or "Revised version" documents, provide reviewer access links. For your "Final submission" document, provide a link to the deposited data.

Files in database submission

Provide a list of all files available in the database submission.

Genome browser session
(e.g. UCSC)

Provide a link to an anonymized genome browser session for "Initial submission" and "Revised version" documents only, to enable peer review. Write "no longer applicable" for "Final submission" documents.

Methodology

Replicates

Describe the experimental replicates, specifying number, type and replicate agreement.

Sequencing depth

Describe the sequencing depth for each experiment, providing the total number of reads, uniquely mapped reads, length of reads and whether they were paired- or single-end.

Antibodies

Describe the antibodies used for the ChIP-seq experiments; as applicable, provide supplier name, catalog number, clone name, and lot number.

Peak calling parameters

Specify the command line program and parameters used for read mapping and peak calling, including the ChIP, control and index files used.

Data quality | *Describe the methods used to ensure data quality in full detail, including how many peaks are at FDR 5% and above 5-fold enrichment.*

Software | *Describe the software used to collect and analyze the ChIP-seq data. For custom code that has been deposited into a community repository, provide accession details.*

Flow Cytometry

Plots

Confirm that:

☒ The axis labels state the marker and fluorochrome used (e.g. CD4-FITC).

☒ The axis scales are clearly visible. Include numbers along axes only for bottom left plot of group (a 'group' is an analysis of identical markers).

☒ All plots are contour plots with outliers or pseudocolor plots.

☒ A numerical value for number of cells or percentage (with statistics) is provided.

Methodology

Sample preparation

FACS: Huh7.5.1. replicon screen cells were washed with phosphate buffered saline (PBS), incubated with 0.05% trypsin for 5 minutes at 37C, then resuspended in Dulbecco's Modified Eagle Medium (DMEM) High glucose supplemented with 10% fetal bovine serum (FBS). Resuspended cells were filtered into round-bottom tubes with cell strainer caps and processed immediately using the SONY sorter.

Flow cytometry: HEK293T or Huh7.5.1. replicon cells were washed with phosphate buffered saline (PBS), incubated with 0.05% trypsin for 5 minutes at 37C, then resuspended in Dulbecco's Modified Eagle Medium (DMEM) High glucose supplemented with 10% fetal bovine serum (FBS). Resuspended cells were analysed immediately using the CytoFLEX.

Intracellular antibody staining: HEK293T parental and replicon cells (300,000 cells per condition) were washed with phosphate buffered saline (PBS), incubated with 0.05% trypsin for 5 minutes at 37C, then resuspended in Dulbecco's Modified Eagle Medium (DMEM) High glucose supplemented with 10% fetal bovine serum (FBS). Cells were fixed and permeabilized using BD Cytofix/Cytoperm kit, incubated with primary antibodies for 30 minutes, incubated with secondary antibody for 20 minutes, resuspended in Perm/Wash buffer then analyzed immediately using the CytoFLEX.
FACS: Huh7.5.1. replicon screen cells were washed with phosphate buffered saline (PBS), incubated with 0.05% trypsin for 5 minutes at 37C, then resuspended in Dulbecco's Modified Eagle Medium (DMEM) High glucose supplemented with 10% fetal bovine serum (FBS). Resuspended cells were filtered into round-bottom tubes with cell strainer caps and processed immediately using the SONY sorter.

Flow cytometry: HEK293T or Huh7.5.1. replicon cells were washed with phosphate buffered saline (PBS), incubated with 0.05% trypsin for 5 minutes at 37C, then resuspended in Dulbecco's Modified Eagle Medium (DMEM) High glucose supplemented with 10% fetal bovine serum (FBS). Resuspended cells were analysed immediately using the CytoFLEX.

Intracellular antibody staining: HEK293T parental and replicon cells (300,000 cells per condition) were washed with phosphate buffered saline (PBS), incubated with 0.05% trypsin for 5 minutes at 37C, then resuspended in Dulbecco's Modified Eagle Medium (DMEM) High glucose supplemented with 10% fetal bovine serum (FBS). Cells were fixed and permeabilized using BD Cytofix/Cytoperm kit, incubated with primary antibodies for 30 minutes, incubated with secondary antibody for 20 minutes, resuspended in Perm/Wash buffer then analyzed immediately using the CytoFLEX.

Instrument | Beckman Coulter CytoFLEX and SONY SH800 Cell sorter

Software | FlowJo software, v10 (Becton Dickinson & Companay)

Cell population abundance | At least 1,000 cells were analyzed per sample.

Gating strategy | Negative control cells were first gated using SSC-A vs FSC-A to exclude cell debris. Then this subset was gated for singlets using FSC-H vs FSC-A. These negative control cells were gated using FITC-A or APC-A histogram to set fluorescence gates. These gates were then propagated to all samples.

☒ Tick this box to confirm that a figure exemplifying the gating strategy is provided in the Supplementary Information.

Magnetic resonance imaging

Experimental design

Design type | *Indicate task or resting state; event-related or block design.*

Design specifications | *Specify the number of blocks, trials or experimental units per session and/or subject, and specify the length of each trial or block (if trials are blocked) and interval between trials.*

Behavioral performance measures | *State number and/or type of variables recorded (e.g. correct button press, response time) and what statistics were used to establish that the subjects were performing the task as expected (e.g. mean, range, and/or standard deviation across subjects).*

Acquisition

Imaging type(s) | *Specify: functional, structural, diffusion, perfusion.*

Field strength | *Specify in Tesla*

Sequence & imaging parameters | *Specify the pulse sequence type (gradient echo, spin echo, etc.), imaging type (EPI, spiral, etc.), field of view, matrix size, slice thickness, orientation and TE/TR/flip angle.*

Area of acquisition | *State whether a whole brain scan was used OR define the area of acquisition, describing how the region was determined.*

Diffusion MRI ☐ Used ☐ Not used

Preprocessing

Preprocessing software | *Provide detail on software version and revision number and on specific parameters (model/functions, brain extraction, segmentation, smoothing kernel size, etc.).*

Normalization | *If data were normalized/standardized, describe the approach(es): specify linear or non-linear and define image types used for transformation OR indicate that data were not normalized and explain rationale for lack of normalization.*

Normalization template | *Describe the template used for normalization/transformation, specifying subject space or group standardized space (e.g. original Talairach, MNI305, ICBM152) OR indicate that the data were not normalized.*

Noise and artifact removal | *Describe your procedure(s) for artifact and structured noise removal, specifying motion parameters, tissue signals and physiological signals (heart rate, respiration).*

Volume censoring | *Define your software and/or method and criteria for volume censoring, and state the extent of such censoring.*

Statistical modeling & inference

Model type and settings | *Specify type (mass univariate, multivariate, RSA, predictive, etc.) and describe essential details of the model at the first and second levels (e.g. fixed, random or mixed effects; drift or auto-correlation).*

Effect(s) tested | *Define precise effect in terms of the task or stimulus conditions instead of psychological concepts and indicate whether ANOVA or factorial designs were used.*

Specify type of analysis: ☐ Whole brain ☐ ROI-based ☐ Both

Statistic type for inference | *Specify voxel-wise or cluster-wise and report all relevant parameters for cluster-wise methods.*

(See Eklund et al. 2016)

Correction | *Describe the type of correction and how it is obtained for multiple comparisons (e.g. FWE, FDR, permutation or Monte Carlo).*

Models & analysis

n/a	Involved in the study
☐	☐ Functional and/or effective connectivity
☐	☐ Graph analysis
☐	☐ Multivariate modeling or predictive analysis

Functional and/or effective connectivity | *Report the measures of dependence used and the model details (e.g. Pearson correlation, partial correlation, mutual information).*

Graph analysis | *Report the dependent variable and connectivity measure, specifying weighted graph or binarized graph, subject- or group-level, and the global and/or node summaries used (e.g. clustering coefficient, efficiency, etc.).*

Multivariate modeling and predictive analysis | *Specify independent variables, features extraction and dimension reduction, model, training and evaluation metrics.*
